# Investigation of Diffusion Characteristics through Microfluidic Channels for Passive Drug Delivery Applications

**DOI:** 10.1155/2016/7913616

**Published:** 2016-05-26

**Authors:** Marcus J. Goudie, Alyssa P. Ghuman, Stephanie B. Collins, Ramana M. Pidaparti, Hitesh Handa

**Affiliations:** College of Engineering, University of Georgia, Athens, GA 30602, USA

## Abstract

Microfluidics has many drug delivery applications due to the ability to easily create complex device designs with feature sizes reaching down to the 10s of microns. In this work, three different microchannel designs for an implantable device are investigated for treatment of ocular diseases such as glaucoma, age-related macular degeneration (AMD), and diabetic retinopathy. Devices were fabricated using polydimethylsiloxane (PDMS) and soft lithography techniques, where surface chemistry of the channels was altered using 2-[methoxy(polyethyleneoxy)propyl]trimethoxysilane (PEG-silane). An estimated delivery rate for a number of common drugs was approximated for each device through the ratio of the diffusion coefficients for the dye and the respective drug. The delivery rate of the model drugs was maintained at a physiological condition and the effects of channel design and surface chemistry on the delivery rate of the model drugs were recorded over a two-week period. Results showed that the surface chemistry of the device had no significant effect on the delivery rate of the model drugs. All designs were successful in delivering a constant daily dose for each model drug.

## 1. Introduction

Various ocular diseases such as age-related macular degeneration (AMD), glaucoma, diabetic retinopathy, and retinitis pigmentosa require lifelong treatment through daily eye drops or monthly injections into the eye to avoid blindness [1]. Due to the cost and frequency of doctor visits, many patients opt to only get injections every 6 months. Decreasing the frequency of injections is detrimental to the patient's eyesight. Monthly injections are required to maintain vision at a constant level or to have any chance of improvement. Ocular diseases are prevalent throughout society, especially affecting adults over the age of 50. An estimated 1.6 million adults suffer from AMD in the US alone, with approximately 500,000 cases diagnosed annually worldwide [2].

Age-related macular degeneration is caused by a buildup of waste in the retina, resulting in blurred central vision and eventual loss of vision. There are two types of AMD: dry and wet. Dry AMD is caused by improper nourishment of the retina, resulting in a buildup of waste in the eye known as drusen. Wet AMD is more threatening, where 90% of cases result in severe vision loss. Breakages in the inner membrane of the retina cause new blood vessels to leak blood and lipid materials into the eye, progressively blurring vision until all sight is lost [3].

Treatment of these ocular diseases is typically done through monthly ocular injections, costing time and money in doctor visits. In addition, the repeated ocular injections run the risks of intraocular infections, hemorrhages, and retinal detachment [4]. While daily eye drops are an alternative to injections, only 5% of the administered drug may reach the anterior intraocular tissues through the cornea [2]. Orally delivered medications may be most convenient for the patient but come with serious systemic side effects due to the high dosages [5]. These high dosages are required for therapeutic levels to be reached in the eye through the blood-retina barrier [6]. Developing an implantable drug delivery device would provide controlled delivery and effective use of drugs, while avoiding required doctor visits and complications from frequent injections. The controlled delivery of drugs will maximize efficiency and allow the patients to experience improvements in their eyesight.

Both biodegradable and nonbiodegradable ocular devices have been approved, each with their own pros and cons. The advantage of biodegradable devices is their ability to degrade after all of the drug has been released. Once they degrade, the device can be replaced to continue delivery of the drug. However, the drug-release lifetime of biodegradable devices can be much shorter than that of nonbiodegradable devices [7]. Nonbiodegradable devices allow for more precise drug release over a longer period of time but remain in the eye after all of the drug has been released and must be replaced.

Currently, there are several options for ocular drug delivery microdevices. Some of the top known devices are from pSivida Corp including Retisert, Iluvien, and Vitrasert. Other top devices in the market include Ozurdex, Surodex, and I-Vation. A biodegradable device example is Ozurdex, whereas Retisert and Iluvien are examples of nonbiodegradable devices. Some of these devices can be inserted into the eye through the use of a needle and others must be surgically implanted in the eye [8]. However, none of the current devices on the market are refillable and must be replaced once the entire drug load has been dispensed.

The field of microfluidics has become a promising tool for drug delivery applications. One of the most common techniques for creating these devices is through the use of soft lithography, where a polymer is poured over a master mold to create the required channels [9, 10]. Polydimethylsiloxane (PDMS) has become the most popular material for these devices due to its inherent biocompatibility and ease of use. Due to the hydrophobic nature of PDMS, methods to alter its surface chemistry have been studied extensively. One of the most common techniques is through the use of oxygen plasma exposure, which provides a dense covering of hydroxyl (–OH) groups on the surface. However, this surface has been shown to recover with time due to migrating uncured oligomers forming the material bulk and rearranging the functional groups away from the surface [11]. It has been shown that these surfaces can be reacted with methoxy-silane or other alkyl-silane groups, by applying a grafted silane to the surface [12, 13]. With PDMS being used for a wide variety of biomedical applications, investigations in altering the surface chemistry of PDMS through grafting of polymer chains or bioactive molecules such as heparin have been studied extensively [14–19].

This study investigates three designs for a refillable microfluidic device for intraocular drug delivery. Various channel configurations were used to demonstrate the effect of surface modification over a range of diffusion lengths, as well as an attempt to limit the burst release kinetics that are associated with reservoir based drug delivery systems. Surface modification of PDMS channels was done through exposure to a silanized polyethylene glycol after exposure to air plasma. Hydrophobic and hydrophilic devices were examined to determine if surface chemistry would alter the drug delivery rate. The release kinetics were studied using two model dyes in various channel geometries over a two-week period.

## 2. Materials and Methods

### 2.1. Materials

For silicon wafer mold fabrication, SU-8 2025 permanent epoxy negative photoresist and SU-8 developer were purchased from MicroChem (Newton, MA). Four-inch silicon wafers were purchased from University Wafer (Boston, MA). Custom laminated masks for patterning of photoresist were designed in AutoCad and sent for printing from CAD/Art Services, Inc. (Bandon, OR). Sylgard 184 silicone elastomer base and curing agent (Dow Corning, Midland, MI) were purchased from Krayden Inc. (Denver, CO). Extra dry acetone, sodium chloride, potassium chloride, sodium phosphate dibasic, potassium phosphate monobasic, Rhodamine B (RB), and Toluidine Blue (TB) were purchased from Sigma Aldrich (St. Louis, MO). Glass slides and cuvettes were purchased from Fisher Scientific. 2-[Methoxy(polyethyleneoxy)propyl]trimethoxysilane (PEG-silane) was purchased from Gelest (Morrisville, PA). Phosphate buffered saline (PBS), pH 7.4, containing 138 mM NaCl, 2.7 mM KCl, 10 mM sodium phosphate, and 100 mM EDTA was used for all experiments.

### 2.2. Design Considerations

**Figure 1:**
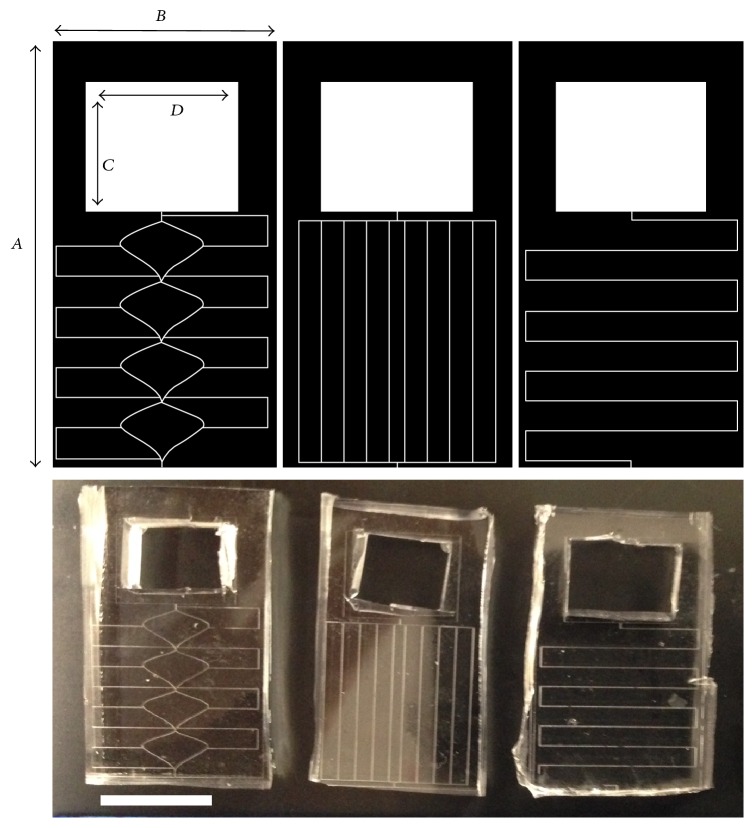
Leaf, Straight, and Snake (left to right) microchannel designs for a range of diffusive characteristics. All channels are 50 *μ*m wide and 50 *μ*m tall. Device length (*A*) 2.5 cm. Width (*B*) 1.5 cm. Reservoir length (*C*) 0.5 cm. Reservoir width (*D*) 0.75 cm. Reservoir height was kept constant at 3 mm. Representative devices for each design are shown below the corresponding graphic (scale bar represents 1 cm).

Three channel configurations, with varying diffusive resistances, are proposed as novel designs for ocular drug delivery and are shown in Figure 1. The large range in resistances was selected to examine if the surface chemistry would have more considerable effects with increasing channel length. The dye is stored in the square reservoir region of the device, where delivery of the drug is done through Fick's second law. The use of the passive devices for drug delivery requires customization for the specific drug as diffusion coefficients of drugs vary and can range within 1.25–3 × 10^−7 ^cm^2^ s^−1^ [21, 23, 24, 26]. Using model dyes with increased diffusion coefficients is beneficial, as measuring extremely dilute solutions can be challenging. A list of drugs and their diffusion characteristics as compared to the model dyes used are shown in Table 1. A number of features of the final device for implantable use were identified including the following:

- Refillable through injection.
- Requiring only minor surgical procedures for implantation.
- Diffusion period between 1 and 2 years.
- Diffusion rate within 2% of the dosing rate.
- Overall volume of less than 280 mm^3^.

The diffusion rates of the three designs were investigated using two dyes as model drugs. The reservoir for each design is identical, totaling 112.5 mm^3^ (3 mm × 5 mm × 7.5 mm, *H* × *L* × *W*). As the dosage requirement and diffusion characteristics vary from drug to drug, the proposed designs are investigated to determine if they are suitable for a variety of deliverable drugs.

### 2.3. Diffusion Modeling

Steady-state diffusion rates were calculated for the three device designs to confirm the experimental measurements. The following assumptions were made for the steady-state analysis: concentration of the dye at the outlet = 0, concentration of the dye in the reservoir is constant, and the delivery of the dye is solely from diffusion governed by Fick's Law (1).

For diffusion-based devices with reservoirs much larger than the channel volumes, along with delivery over a long period of time, diffusion can be approximated using Fick's first law, relating the diffusive flux to the concentration gradient:

(1) $$\begin{array}{c} J=-D\frac{\partial C}{\partial x}, \end{array}$$

where *J* is the diffusive flux (mol m^−2^ s^−1^), *D* is the diffusion coefficient (m^2^ s^−1^), and *C* is the concentration of drug in the reservoir (mol m^−3^). The molar delivery of the drug (*M*) into the eye is then

(2) $$\begin{array}{c} M=JA, \end{array}$$

where *A* is the cross sectional area of the microfluidic channel (m^2^).

For steady-state, the flux can be simplified to the change in concentration times the diffusive resistance. For the Snake design, the resistance was taken to be the inverse of the channel length (m^−1^), whereas the resistance for the Straight and Leaf designs was calculated using the analogous method for parallel resistors in a circuit:

(3) $$\begin{array}{c} R_{eq}=\underset{i}{\sum }\frac{1}{L_{i}}. \end{array}$$

Desired molar delivery rates for the example drugs were calculated (nmol/day) and are shown in Table 1. To correlate the model drug dyes to a predicted delivery rate of the proposed drug, the flux is multiplied by the ratio of the diffusion coefficients between the two compounds and the ratios of the concentrations to be used in the reservoirs:

(4) $$\begin{array}{c} M_{drug}=\frac{D_{drug}}{D_{dye}}J_{dye}A. \end{array}$$

The diffusion coefficient used for Rhodamine B has been reported previously through experimental testing [25], while the coefficient for Toluidine Blue was approximated using the Stokes-Einstein approximation.

### 2.4. Device Fabrication

Devices were created using soft lithography techniques. Negative photoresist SU-8 2025 was spin coated on silicon wafers to achieve a layer of 50 microns. Wafers were exposed to UV light (365 nm) using a Karl Suss MA6 mask aligner with soft contact. Preexposure bake, exposure time, and postexposure bake were all determined from the MicroChem datasheet. A 10 : 1 ratio of PDMS base and a curing agent was mixed thoroughly, poured over the silicon wafer, and degassed in a vacuum for 1 hour. The PDMS was then left in an oven at 90°C for 60 minutes. After curing, the PDMS was peeled away from the molds forming the channels. The individual devices were cut out and the reservoir region was removed.

**Figure 2:**
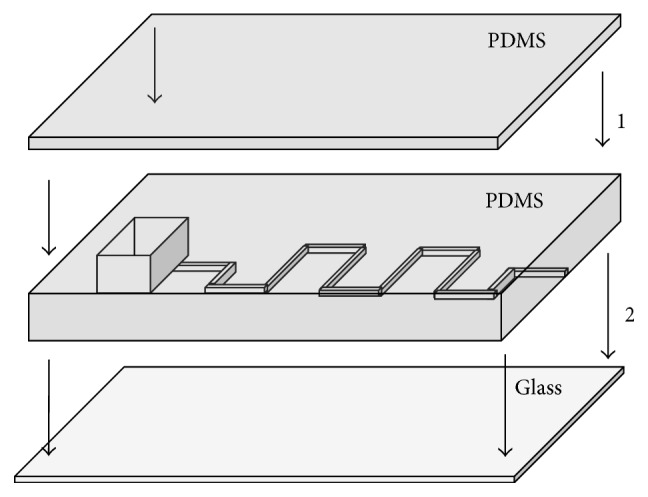
Bonding procedure for device fabrication. (1) Top PDMS is bonded to seal top of the reservoir. (2) PDMS channel design is bonded to glass slide. Devices for hydrophilic surface chemistry were filled immediately after step (2).

Bonding of the devices was done through exposure to air plasma (Harrick Plasma Cleaner) in a two-step process (Figure 2): (1) a top section of PDMS was bonded to the PDMS block with the channels and (2) channel side of PDMS was bonded to a glass base. The side to be bonded was exposed to oxygen plasma (700 mTorr air) for 5 minutes and subsequently brought into contact with the glass slide or PDMS in order to form an irreversible seal [27]. After plasma treatment, hydrophobic devices were left on a hot plate at 80°C for 30 minutes. Devices were left to sit for one week to ensure hydrophobic recovery of the channel walls prior to testing [11]. For devices to have hydrophilic surfaces, channels were filled with a 50 : 50 solution of PEG-silane and extra dry acetone and allowed to react for 1 hour at room temperature after exposure to oxygen plasma [12, 13]. Hydrophilic devices were then rinsed thoroughly with PBS and flushed with air to dry.

### 2.5. Confirmation of PEG Attachment

Contact angles of untreated PDMS and PEG treated PDMS were measured to validate the method used to change the surface chemistry for the devices. Films of PDMS (10 : 1 base to curing agent) were poured, degassed for 1 hour, and cured at 80°C for 1 hour. Films to be plasma treated were then exposed to air plasma for 5 min in the Harrick Plasma Cleaner. Following plasma treatment, films to be exposed to the PEG-silane were immediately submerged in the 50 : 50 PEG-silane: extra dry acetone solution for one hour. After one hour, films were rinsed thoroughly with deionized water and left to dry for 24 hours. Contact angle was measured at three randomly selected areas for each sample (*N* = 3) using a Kruss Drop Shape Analyzer.

### 2.6. UV Spectra of Rhodamine B and Toluidine Blue

Full spectra of both RB and TB were measured to confirm adsorption peaks as found in the literature using a Cary Bio Spectrophotometer (Varian). Scans were recorded from 900 to 200 nm. Baseline measurements were measured with 0.1 M PBS.

### 2.7. *In Vitro* Testing

The three channel configurations were tested using the two dyes as model drugs, where concentration of the dye in the external reservoir was measured. Smallest diffusion path was varied between the device designs from 15 mm to 115 mm. Height and width of the microfluidic channels were kept constant between the devices at 50 *μ*m.

**Figure 3:**
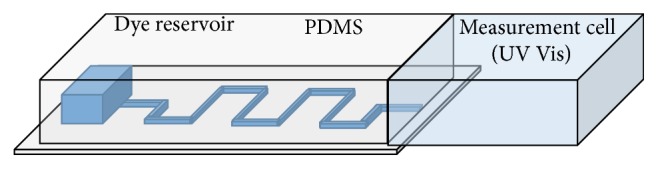
Representation of experimental setup for measurement of total dye diffused from the microchannel device.

Diffusion characteristics of the devices were measured by filling the reservoirs of the devices with either 5 mM Rhodamine B or 32 mM Toluidine Blue using a 1 mL syringe with 27 gauge needle. These concentrations were selected as they are near the saturation limit of the model drugs. After devices were filled, they were placed in a sealed plastic centrifuge tube with 4 mL of (PBS) and placed in a Series 2 Water Jacketed Incubator (Thermo Scientific) at 37°C to simulate the vitreous humor of the eye. Absorbance values of the solution of PBS and dye for each device were measured using Genesys 10S UV-Vis Spectrophotometer (Thermo Scientific) every 24 hours at 550 nm [28] and 640 nm [29] for Rhodamine B (RB) and Toluidine Blue (TB), respectively. The device was taken out of the centrifuge tube and the liquid was mixed in a vortex mixer before each measurement. Concentration of the model drug in the PBS was calculated using a calibration curve for the corresponding dye, which was created using five known concentrations. A representation of the test configuration is shown in Figure 3.

## 3. Results and Discussion

### 3.1. Steady-State Analysis

Delivery rate of each dye for each design was calculated as a way to assess the validity of each experimental data set. While the burst release of these devices is important to consider for patient safety, it is also important to consider the clinically relevant concentration for the drugs to be active at the delivery site. Likewise, if the release rate is high for one day when devices are to be used on the order of years, this increased release rate can be insignificant in the overall effectiveness of the device. Resistance to diffusion was calculated for each design using (3), using the sum of the inverse of the path lengths, along with the theoretical delivery rate of each dye (Table 2). The steady-state delivery rates for the drugs listed in Table 1 are also shown. The target lifetime for these devices is between 1 and 2 years.

### 3.2. Confirmation of PEG Attachment

**Figure 4:**
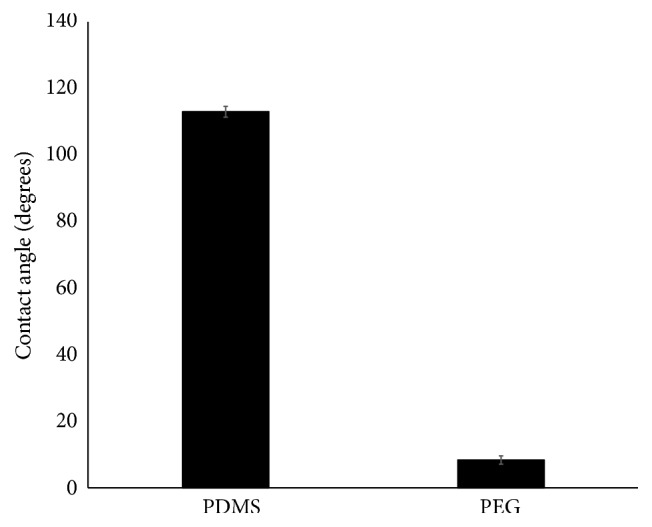
Confirmation of PEG attachment to PDMS as measured through contact angle. Measurement was conducted 24 hours after air plasma exposure.

The method to modify the PDMS surface was confirmed through contact angle measurements. After air plasma exposure, PDMS to be treated were submerged in 50 : 50 PEG-silane:extra dry acetone for one hour. After thoroughly rinsing with deionized water, films were left to sit 24 hours prior to measurement due to the recovery of PDMS after plasma exposure. Contact angle was observed to decrease from 113 ± 1.63° (PDMS) to 8.33 ± 1.24° (PEG) and is shown in Figure 4. This significant decrease in contact angle confirms not only the attachment of the PEG-silane to the surface, but also the fact that the attachment is highly uniform as very little deviations are seen throughout the surface, but between samples as well.

### 3.3. UV Spectra of Rhodamine B and Toluidine Blue

**Figure 5:**
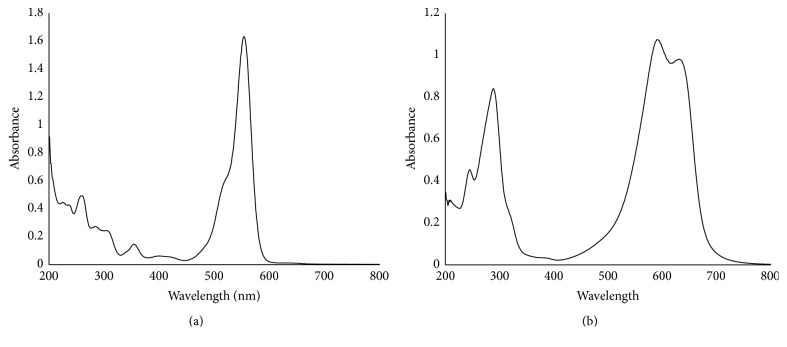
Full UV spectra for (a) Rhodamine B and (b) Toluidine Blue.

Full UV spectra of both RB and TB were shown to have peaks similar to those reported in the literature [28, 29]. Rhodamine B was found to have a peak at 550 nm, where TB has two peaks in the 600 region (596 nm and 640 nm). Full spectra of both RB and TB are shown in Figure 5.

### 3.4. *In Vitro* Testing

**Figure 6:**
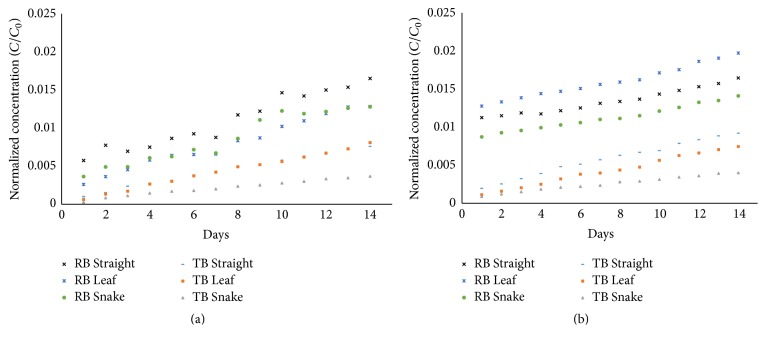
Normalized outlet concentration of Rhodamine B and Toluidine Blue as measured by UV-Vis spectrophotometer. (a) Release from hydrophilic devices. (b) Release from hydrophobic devices.

The three channel geometries (Straight, Snake, and Leaf) were tested* in vitro* over a two-week period. While the PBS in the measurement cell of the device may not replicate the ocular fluid directly, the rate limiting step in consumption of the drug stems from the diffusion of the drug from the devices from the channel design, and not the diffusion of the drug from exit of the device to the final treatable area. Therefore, we feel that the measurement technique is valid for measuring cumulative delivery of the drug from each design. As with diffusion-limited delivery methods, a burst release was seen in the first 24 hours. After 24 hours, the devices reached steady-state delivery. The normalized amount of delivered model drugs for each design with each surface chemistry is shown in Figure 6, where *C*_0_ denotes the initial concentration of the model drug in the device reservoir. As the diffusion of the dyes reached steady-state after 24 hours, the diffusion rates were calculated for both stages of the device (first 24 hours, after 24 hours). Delivery rates for the two stages of delivery are summarized in Table 3. Normalized release rates for both model drugs were examined for both hydrophilic and hydrophobic channels. While there are higher initial releases for all designs, release rates are on the same order of magnitude for three of the four device conditions. Hydrophilic devices as measured with RB showed considerable burst and may be from excess pressure in the reservoir when priming the devices, as the PDMS top layer is thin. The deformation of the top layer can provide an active pumping stage, which may be beneficial for applications requiring immediate relief. As the primary focus of this paper is to observe passive delivery, the ability for these devices to provide active pumping is outside of the scope of this paper and is being further investigated.

The steady-state delivery of each model drug was compared to the theoretical steady-state value for each design (Table 4). The decreased delivery rate for the experimental testing can be attributed to the permeable nature of PDMS, as the channel walls were not solid boundaries as assumed in the steady-state analysis. This can be confirmed through the observation that the effective Toluidine Blue delivery was a lower percentage than the Rhodamine B delivery rate, as the smaller TB dye can diffuse through the PDMS more easily. While this effect will be minimized when using large molecule drugs, some treatments such as the fluocinolone acetonide (MW 452 g/mol) are small enough that these diffusion rates in the PDMS devices are not negligible. Controlling the delivery rate in future designs for diffusion-based drug delivery systems utilizing PDMS should primarily utilize decreasing channel dimensions as opposed to increasing channel lengths to limit drug release.

To correlate the delivery rates seen by the model dyes, rates of the proposed drugs were estimated by multiplying the delivery rate by the ratio of the diffusion coefficients. A table of the estimated drug delivery values is shown in Table 5. Hydrophobic Straight and Leaf designs were best suited for Lucentis and Intravitreal Avastin but did not meet the dosing accuracy of ±2% of the target dose. Currently, scaling of these devices is being explored for implantable devices and will be tested in the future.

## 4. Conclusions

Three microfluidic device designs were tested* in vitro* using two model drugs and compared to theoretical delivery rates. Three channel configurations were proposed to provide a wide range of drug delivery rates, while also examining the effect of surface wettability on delivery of these drugs. The devices were fabricated using soft lithography techniques and tested over a two-week period using Rhodamine B and Toluidine Blue as model drugs. Confirmation of PEG surface modification was observed through the decrease in contact angle from 113 ± 1.63° (PDMS) to 8.33 ± 1.24° (PEG). As observed previously from diffusion-controlled drug delivery systems, delivery rates from all designs provided a burst release during the first 24 hours and then reached a steady-state for the following 13 days. It was observed that altering the surface chemistry of these channels had no significant effect on the release kinetics of the model drugs from the devices, as well as not limiting the adsorption of the model drugs into the bulk device. An estimated delivery rate for a number of common drugs was approximated for each device through the ratio of the diffusion coefficients for the dye and the respective drug. The Straight and Leaf designs most closely matched the required dosages of Lucentis and Intravitreal Avastin. However, all three designs provided a constant delivery of the model drugs after 24 hours. Based on these results, scaling of these devices for an implantable model is currently under investigation.

## Figures and Tables

**Table 1:** Dosing and diffusion characteristics of common drugs to treat ocular diseases. Diffusion coefficient of Toluidine Blue calculated using Stokes-Einstein approximation.

Drug name (type)	Diffusion coefficient (cm^2^/s)	Average dosage (nL/min)	Injection amounts/periods	Molecular weight	Molar delivery (nmol/day)	References
Macugen (pegaptanib sodium)	3 × 10^−7^	0.2083	3 mg/10 days	400 kDa	6.00	[20]
Lucentis (ranibizumab)	2.08 × 10^−7^	0.0124	0.5 mg/month	500 kDa	0.35	[21]
Intravitreal Avastin (bevacizumab)	1.25 × 10^−7^	0.0289	1.25 mg/month	100 kDa	0.28	[22]
Fluocinolone Acetonide	2.3 × 10^−7^	0.0744	15 mg/20 weeks	400 Da	237.04	[23, 24]

Toluidine Blue	1.42 × 10^−5^					
Rhodamine B	4.2 × 10^−6^					[25]

**Table 2:** Steady-state delivery rates for the various channel geometries as compared to the desired dosing rate.

Drug	Design	Flux mol m^−2^ s^−1^	nmol/day	Desired dosing rate (nmol/day)	Lifetime (years)
Macugen	Snake	2.68*E* − 06	0.579	6	1.042
	Leaf	1.71*E* − 05	3.703		0.163
	Straight	6.11*E* − 05	13.207		0.046

Lucentis	Snake	1.86*E* − 06	0.401	0.3472	1.503
	Leaf	1.19*E* − 05	2.567		0.235
	Straight	4.24*E* − 05	9.157		0.066

Intravitreal Avastin	Snake	1.12*E* − 06	0.241	0.279	2.500
	Leaf	7.14*E* − 06	1.543		0.391
	Straight	2.55*E* − 05	5.503		0.110

Fluocinolone acetonide	Snake	2.05*E* − 06	0.444	237	1.359
	Leaf	1.31*E* − 05	2.839		0.212
	Straight	4.69*E* − 05	10.125		0.060

**Table 3:** Normalized delivery rates for first 24 hours and following 13 days for each design and surface chemistry.

Device	Design	Day 1 (*C*/*C*_0_ per day)	13-day average (*C*/*C*_0_ per day)
TB	Straight	1.25*E* − 01	3.56*E* − 02
	Leaf	7.22*E* − 02	3.16*E* − 02
	Snake	5.62*E* − 02	1.52*E* − 02

TB-PEG	Straight	6.44*E* − 02	3.22*E* − 02
	Leaf	4.00*E* − 02	3.52*E* − 02
	Snake	1.80*E* − 02	1.56*E* − 02

RB	Straight	8.98*E* − 05	1.25*E* − 05
	Leaf	4.06*E* − 05	1.25*E* − 05
	Snake	5.67*E* − 05	1.25*E* − 05

RB-PEG	Straight	1.76*E* − 04	6.25*E* − 06
	Leaf	1.99*E* − 04	6.25*E* − 06
	Snake	1.36*E* − 04	6.25*E* − 06

**Table 4:** Comparison of measured* in vitro* rates to theoretical delivery rates for all geometries.

Device	Design	13 day measured average (*C*/*C*_0_ per day)	Theoretical steady state approximation (SSA)	Average/SSA
TB	Straight	0.0713	0.7742	0.0921
	Leaf	0.0622	0.6636	0.0938
	Snake	0.0307	0.1037	0.2962

TB-PEG	Straight	0.0649	0.7742	0.0838
	Leaf	0.0735	0.6636	0.1108
	Snake	0.0334	0.1037	0.3217

RB	Straight	0.0012	0.003	0.3939
	Leaf	0.0014	0.0026	0.5385
	Snake	0.0018	0.0004	4.5263

RB-PEG	Straight	0.0009	0.003	0.2934
	Leaf	0.0011	0.0026	0.4129
	Snake	0.0008	0.0004	2.0449

**Table 5:** Fraction of estimated delivered drug to required dosing rate for each design for commonly used drugs. Ratio is given by delivery drug/recommended dose.

Device	Design	Macugen	Lucentis	Intravitreal Avastin	Fluocinolone acetonide
TB	Straight	0.108	1.292	0.964	0.002
	Leaf	0.096	1.146	0.855	0.002
	Snake	0.046	0.551	0.411	0.001

TB-PEG	Straight	0.098	1.168	0.872	0.002
	Leaf	0.107	1.277	0.953	0.002
	Snake	0.047	0.566	0.422	0.001

RB	Straight	23.81	285.00	212.00	0.462
	Leaf	23.81	285.26	212.86	0.462
	Snake	23.81	285.26	212.86	0.462

RB-PEG	Straight	23.81	285.26	212.86	0.462
	Leaf	23.81	285.26	212.86	0.462
	Snake	23.81	285.26	212.86	0.462
